# Left ventricular hypertrophy, diastolic dysfunction and right ventricular load predict outcome in moderate aortic stenosis

**DOI:** 10.3389/fcvm.2022.1101493

**Published:** 2023-01-10

**Authors:** Stephan Stöbe, Joscha Kandels, Michael Metze, Bhupendar Tayal, Ulrich Laufs, Andreas Hagendorff

**Affiliations:** ^1^Klinik und Poliklinik für Kardiologie, Universitätsklinikum Leipzig, Leipzig, Germany; ^2^Methodist DeBakey Heart and Vascular Center, Houston, TX, United States

**Keywords:** valvular heart disease, aortic stenosis (AS), echocardiography, outcome, longitudinal strain

## Abstract

**Aims:**

Predictors of progression of moderate aortic valve stenosis (AS) are incompletely understood. The objective of this study was to evaluate the prognostic value of left ventricular hypertrophy (LVH), diastolic dysfunction, and right ventricular (RV) load in moderate AS.

**Methods and results:**

Moderate AS was defined by aortic valve area (AVA), peak transvalvular velocity (V_max_) or mean pressure gradient (PG_mean_). A total of 131 Patients were divided into two groups according to the number of pathophysiological changes (LVH, diastolic dysfunction with increased LV filling pressures and/or RV load): <2 (group 1); ≥2 (group 2). The primary outcome was survival without aortic valve replacement (AVR). After follow-up of 30 months, the reduction of AVA (–0.06 ± 0.16 vs. –0.24 ± 0.19 cm^2^, *P* < 0.001), the increase of PG_mean_ (2.89 ± 6.35 vs 6.29 ± 7.13 mmHg, *P* < 0.001) and the decrease of the global longitudinal strain (0.8 ± 2.56 vs. 1.57 ± 3.42%, *P* < 0.001) from baseline to follow-up were significantly more pronounced in group 2. Survival without AVR was 82% (group 1) and 56% (group 2) [HR 3.94 (1.74–8.94), *P* < 0.001]. Survival without AVR or progression of AS was 77% (group 1) and 46% (group 2) [HR 3.80 (1.84–7.86), *P* < 0.001]. The presence of ≥2 pathophysiological changes predicted outcome whereas age, comorbidities, LDL-cholesterol did not.

**Conclusion:**

The presence of ≥2 pathophysiological changes is a strong predictor of outcome in moderate AS and may be useful for risk stratification, particularly for scheduling follow-up time intervals and deciding the timing of AVR.

## Introduction

Patients with severe symptomatic aortic stenosis (AS) have a poor prognosis and benefit from aortic valve replacement (AVR) (1–3). In contrast, the natural history and clinical outcome of patients with moderate AS are incompletely described and parameters for risk stratification are sparse. Moderate AS is defined by echocardiography on the basis of aortic valve area (AVA), peak transvalvular velocity (V_max_) and mean pressure gradient (PG_mean_) (3, 4). The importance of V_max_, PG_mean_, and valve calcification on clinical outcome is not clear (5–8). Most of the studies on progression of moderate AS are limited by small study populations, do not include echocardiographic assessment and/or were performed 20–30 years ago (9).

There is no specific therapy available to prevent moderate AS progression. The treatment focuses on the prevention of atherosclerosis including optimal treatment of comorbidities and risk factors. Patients with moderate AS are recommended to be regularly monitored by echocardiography but individual AS progression varies widely causing difficulties to implement general recommendations of risk stratification (3, 8).

Considering the pathophysiology of AS, left ventricular hypertrophy (LVH), diastolic dysfunction (DD) with increased LV filling pressures, and/or right ventricular (RV) load are characteristics of patients with advanced stages of AS. Recently, we assessed the presence of LVH, DD (E/e’), and RV load (maximum tricuspid regurgitation velocity, TR_Vmax_) in patients with severe AS (10). We observed that severe AS is highly unlikely without the presence of LVH including normal values for E/e’ and TR_Vmax_ (10).

Based on these findings we hypothesized that LVH, E/e’ and TR_Vmax_ may have a prognostic value in moderate AS.

## Materials and methods

### Study population

Between 2016 and 2019 patients with moderate AS meeting the following inclusion criteria were included: AVA 1.0–1.5cm^2^ and V_max_ > 2.9 m/s (<4.0 m/s), or PG_mean_ > 20 mmHg (<40 mmHg) (3, 4). Patients with concomitant moderate or severe valvular disease, already confirmed cardiac amyloidosis, hypertrophic obstructive cardiomyopathy, acute myocarditis, LV ejection fraction (EF) <45% or/and LV stroke volume index (SVi) <35 ml/m^2^, pulmonary hypertension due to pulmonary disease and/or acute pulmonary embolism, body mass index ≥35 kg/m^2^, prior heart surgery or valvular intervention were excluded. The prospective study was conducted in accordance with the Declaration of Helsinki and was approved by the ethical committee of the University of Leipzig (041/19-ek). All included patients provided informed consent.

Clinical follow-up was available in 131 of 157 patients. All patients were monitored clinically and by echocardiography every 6 months. The inclusion date corresponds to the date of baseline transthoracic echocardiography (TTE). Transesophageal echocardiography (TEE) was performed in cases of uncertain findings by TTE. Moderate AS patients were divided into two groups according to the number of pathophysiological changes [LVH: increased left ventricular mass index (LVMi), DD: increased E/e’ and/or RV load: increased TR_Vmax_]: group 1: <2 changes (*n* = 79); group 2: ≥2 changes (*n* = 52). The primary outcome was survival without AVR. The secondary outcome was survival without AVR or progression from moderate to severe AS based on current recommendations (3). Clinical decisions on referral for AVR were made by heart team decisions.

Patient characteristics were collected from the patients and from medical records. At baseline, all patients were asymptomatic or presented with unspecific and/or only mild symptoms. N-terminal pro-B-type natriuretic peptide (NT pro-BNP), low-density lipoprotein-(LDL)-cholesterol, and lipoprotein(a) [Lp(a)] (cut-off value of >58.5 mg/dl/140 nmol/l) were assessed (11).

### Echocardiography

Transthoracic echocardiography was performed using a Vivid E9 or E95 ultrasound system with a M5-S or a 4Vc phased array probe and echocardiographic analyses were performed with the EchoPac software version 203 or 204 (GE Healthcare Vingmed Ultrasound AS, Horten, Norway).

### Evaluation of aortic valve stenosis

Effective AVA was calculated by the continuity equation. The diameter of the left ventricular outflow tract (D_LVOT_) was determined by TTE in the parasternal long axis view in the LVOT at a distance of 5–10 mm from the aortic valve during mid-systole. Only in a few exceptional cases D_LVOT_ was measured by TEE in the mid-esophageal long axis view. LVOT blood flow velocities were assessed by pulsed wave (PW) Doppler echocardiography in the apical long axis view by placing the sample volume at the position corresponding to the position of D_LVOT_ measurements. V_max_ was determined by continuous wave (CW) Doppler echocardiography either in the apical long axis or 5-chamber view. PG_mean_ was calculated by the (simplified: if pre-stenotic velocities were in normal ranges) Bernoulli equation (3, 4). Progression from moderate to severe AS was assessed by AVA, V_max_ and PG_mean_ by experienced cardiologists based on current recommendations (3, 4).

### Left ventricular volumes/function and pathophysiological changes

left ventricular hypertrophy was defined by LVMi (males: ≥115 g/m^2^; females: ≥95 g/m^2^) using the Devereux formula (12, 13). LV mass was assessed by M-Mode measurements using parasternal short axis views. LV volumes and LVEF were assessed by biplane LV planimetry using the modified Simpson’s rule (13). Myocardial deformation was characterized by global longitudinal peak systolic strain (GLS) by 2D speckle tracking analysis in the apical long axis-, 2- and 4-chamber-view (14, 15). Endocardial contours and tracking areas were adjusted manually to enable full myocardial tracking. Only segments with accurate tracking were accepted.

Valvulo-arterial impedance (Z_VA_) was calculated by PG_mean_, systolic blood pressure (sBP) and LVSVi by the following equation: Z_VA_ = (PG_mean_ + sBP)/LVSVi (16). sBP was measured in supine position at the time of TTE using an automatic arm-cuff blood pressure monitor.

Diastolic dysfunction was assessed according to current recommendations (17). Mild DD in terms of LV relaxation disorder was not considered sufficient, so relevant DD was defined by DD with an increase of LV filling pressures (increased E/e’): ≥14 (sinus rhythm) or ≥11 (atrial fibrillation, AF) (17).

Right ventricular load was defined by an increase of TR_Vmax_ ≥2.8 m/s in the apical 4-chamber-view (17). RV systolic function was evaluated by tricuspid annular plane systolic excursion (TAPSE). Normal RV function was defined by TAPSE >17 mm (13).

### Statistical analysis

Statistical analyses were performed using SPSS Statistics (version 24.0, IBM, Armonk, NY, USA). Kolmogorov–Smirnov was used to test normal data distribution. Continuous variables are expressed as mean ± standard deviation (SD) and differences between two groups were analyzed by student’s *t*-test. Follow-up period was expressed by median ± interquartile range. All categorical variables were expressed as numbers and/or percentages. Chi-squared or Fisher’s exact test were used to analyze categorical variables as appropriate. Kappa coefficient (κ) was used to assess interrater reliability for LVH, E/e’ and TR_Vmax_ in 20 randomly selected patients. Kaplan-Meier time-to-event analyses were performed and compared by log-rank test. Multivariate analysis was done by cox proportional-hazards model. A *P*-value < 0.05 was considered to indicate statistical significance.

## Results

Baseline characteristics were balanced between both groups, except for a higher percentage of patients with coronary heart disease (CHD) in group 2 and a slightly higher percentage of patients with bicuspid AS in group 1 (Table 1).

**TABLE 1:** Baseline characteristics of patients with moderate aortic valve stenosis (AS).

Variables	All patients (*n* = 131)	Group 1: <2 (*n* = 79)	Group 2: ≥2 (*n* = 52)	*P*-value
Age, years	72 ± 9.90	72 ± 9.38	73 ± 9.31	0.79
Female	43 (33%)	25 (32%)	18 (35%)	0.89
BMI, kg/m^2^	28.56 ± 5.09	28.29 ± 4.71	28.73 ± 5.08	0.43
sBP, mmHg dBP, mmHg	140 ± 17 77 ± 11	141 ± 17 79 ± 11	139 ± 19 75 ± 11	0.20 0.08
Hypertension	103 (79%)	61 (77%)	42 (81%)	0.62
Diabetes mellitus	44 (34%)	26 (33%)	18 (34%)	0.84
Hypercholesterolemia	66 (51%)	42 (53%)	24 (47%)	0.44
Peripheral vascular disease	14 (11%)	7 (9%)	7 (13%)	0.43
CHD	47 (36%)	23 (29%)	24 (46%)	**<0.05**
Bicuspid valve	8 (6%)	6 (8%)	2 (3%)	**<0.05**
Atrial fibrillation	35 (27%)	21 (26%)	14 (27%)	0.97
Stroke	23 (18%)	15 (19%)	8 (15%)	0.59
COPD	9 (7%)	5 (7%)	4 (8%)	0.78
Smoker	37 (28%)	24 (30%)	13 (26%)	0.52
CKD ≥ 3	53 (40%)	30 (38%)	23 (45%)	0.92
NYHA	1.5 ± 0.6	1.5 ± 0.6	1.5 ± 0.6	0.28
Angina pectoris	19 (14%)	12 (15%)	7 (13%)	0.23
Previous syncope	5 (4%)	4 (5%)	1 (2%)	0.33
Statins	86 (66%)	50 (63%)	36 (69%)	0.79
LDL-Cholesterol, mmol/l	2.96 ± 1.02	2.94 ± 0.95	2.98 ± 1.15	0.84
NT pro-BNP, pg/ml	470 ± 251	386 ± 233	695 ± 284	0.20

Data are expressed as mean ± SD or as *n* (%), *P*-value < 0.05 (bold) was considered to indicate statistical significance. BMI, body mass index; s/dBP, systolic/diastolic blood pressure; CHD, coronary heart disease; COPD, chronic obstructive pulmonary disease; CKD, chronic kidney disease; NYHA, New York Heart Association; LDL, low-density lipoprotein; NT pro-BNP, N-terminal pro-B-type natriuretic peptide.

### Echocardiographic parameters

An increase of LVMi was the most common echocardiographic finding in both groups (group 1: 48%; group 2: 92%), followed by increase of TR_Vmax_ (group 1: 14%; group 2: 69%) and E/e’ (group 1: 6%; group 2: 63%). Based on this classification, LVMi, E/e’ and TR_Vmax_ were higher in group 2 (*P* < 0.001^†, ‡^). However, a significant increase between baseline and follow-up was only observed for LVMi (both groups, *P* < 0.05*) and TR_Vmax_ (group 2, *P* < 0.05*; Table 2). Interrater variability revealed high agreement for LVH, E/e’, and TR_Vmax_ (LVH: κ = 0.74 (z = 3.42, *P* < 0.001); E/e’: κ = 0.90 (z = 3.66, *P* < 0.001); TR_Vmax_ κ = 0.80 (z = 3.66, *P* < 0.001).

**TABLE 2:** Echocardiographic results of patients with moderate aortic valve stenosis (AS).

Variables	Group 1: <2 (*n* = 79)			Group 2: ≥2 (*n* = 52)		
	Baseline	Follow-up	*P*-value	Baseline	Follow-up	*P*-value
AVA, cm^2^	1.21 ± 0.16*	1.16 ± 0.18*^‡^	**<0.05** *	1.23 ± 0.15*	1.00 ± 0.21*^‡^	<0.001* 0.28^†^ **<0.001**^‡^
PG_mean_, mmHg	24.45 ± 6.11*	27.14 ± 6.84*^‡^	**<0.05** *	25.41 ± 6.99*	31.74 ± 9.21*^‡^	< 0.001* 0.55^†^ **< 0.05**^‡^
LVMi, g/m^2^	105.98 ± 16.90*^†^	110.52 ± 17.63*^‡^	**<0.05** *	124.14 ± 17.43*^†^	132.72 ± 18.63*^‡^	<0.05* **<0.001**^†^ **<0.001**^‡^
E/e’	10.72 ± 2.44^†^	11.37 ± 3.39^‡^	0.11	16.01 ± 4.09^†^	17.07 ± 4.87^‡^	0.09* **<0.001**^†^ **<0.001**^‡^
TR_Vmax_, m/s	2.63 ± 0.35^†^	2.69 ± 0.30^‡^	0.06	2.97 ± 0.32*^†^	3.16 ± 0.40*^‡^	<0.05* **<0.001**^†^ **<0.001**^‡^
LVEF, %	59.70 ± 5.36	59.62 ± 5.27	0.26	59.80 ± 5.83	59.37 ± 5.96	0.76* 0.95^†^ 0.82^‡^
GLS, %	–18.59 ± 3.12*	–17.76 ± 2.94*^‡^	**<0.05** *	–17.71 ± 3.35*	–16.14 ± 3.91*^‡^	<0.05* 0.18^†^ **<0.05**^‡^
Z_*VA*,_ mmHg/ml/m^2^	3.96 ± 0.92	3.76 ± 0.79	0.14	3.95 ± 0.72	4.01 ± 0.80	0.38* 0.94^†^ 0.09^‡^
TAPSE, mm	20.51 ± 3.92	20.25 ± 3.66	0.55	20.64 ± 3.85	19.80 ± 3.26	0.26* 0.86^†^ 0.51^‡^

Data are expressed as mean ± SD or as *n* (%), *P*-value < 0.05 (bold) was considered to indicate statistical significance (*statistically significant between baseline and follow-up, ^†^statistically significant between baseline group 1 and baseline group 2, ^‡^statistically significant between follow-up group 1 and follow-up group 2). AVA, aortic valve area; PG, pressure gradient; LVMi, left ventricular mass index; TR, tricuspid regurgitation; V_max_, maximum velocity; LVEF, left ventricular ejection fraction; GLS, global longitudinal strain; Z_VA_, Valvulo-Arterial impedance; TAPSE, tricuspid annular plane systolic excursion.

AVA and PG_mean_ were similar at baseline. After 30 months of follow-up (30 ± 5 months) AVA was significantly lower and PG_mean_ was significantly higher in both groups. Changes in AVA (*P* < 0.001^‡^) and PG_mean_ (*P* < 0.05^‡^) were more pronounced in patients with ≥2 pathophysiological changes (Table 2).

Almost all patients had a LVEF >50% (95%; 5% had an LVEF between 45 and 49%) and a normal TAPSE (100%) at baseline and follow-up (Table 2). GLS was significantly lower at follow-up, which was even more pronounced in group 2 (group 1: Δ –0.83 ± 0.18 vs. group 2: Δ –1.72 ± 0.04, *P* < 0.05^‡^; Table 2). Z_VA_ did not differ between both groups neither at baseline nor at follow-up (Table 2).

### Predictors of outcome

**FIGURE 1:**
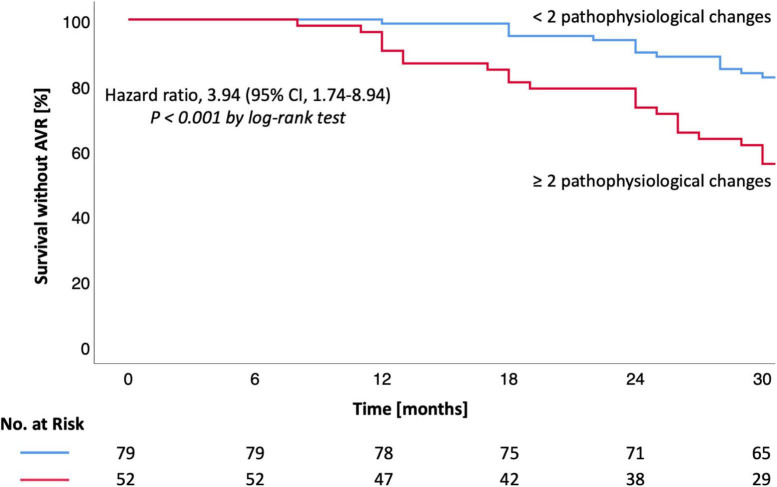
Kaplan-Meier estimates for survival without AVR in moderate AS. Kaplan-Meier estimates are shown for the primary outcome (survival without AVR). CI denotes confidence interval. Hazard ratio and CI were calculated by cox proportional-hazards model (log-rank test). A *P*-value < 0.05 was considered to indicate statistical significance. AS, aortic valve stenosis; AVR, aortic valve replacement.

**FIGURE 2:**
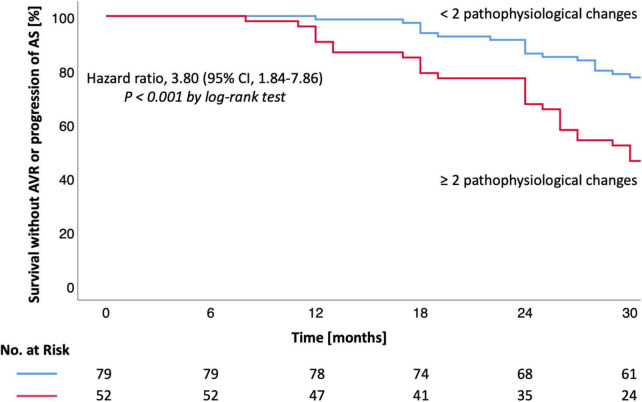
Kaplan-Meier estimates for survival without AVR or AS progression in moderate AS. Kaplan-Meier estimates are shown for the secondary outcome (survival without AVR or AS progression). CI denotes confidence interval. Hazard ratio and CI were calculated by cox proportional-hazards model (log-rank test). A *P*-value < 0.05 was considered to indicate statistical significance. AS, aortic valve stenosis; AVR, aortic valve replacement.

Survival without AVR was 99% at 12, 90% at 24, and 82% at 30 months (group 1) vs. 90, 73, and 56% (group 2) [HR 3.94 (1.74–8.94), *P* < 0.001, Table 3 and Figure 1]. Survival without AVR or progression of AS was 99% at 12, 86% at 24, and 77% at 30 months (group 1) vs. 90, 67, and 46% (group 2) [HR 3.80 (1.84–7.86), *P* < 0.001, Table 3 and Figure 2].

**TABLE 3:** Outcomes in moderate aortic stenosis (AS).

Outcome	Group 1: <2 (*n* = 79)	Group 2: ≥2 (*n* = 52)	HR (95% CI)	*P*-value
	No. (%)	No. (%)		
**Primary outcome**				
Survival without AVR	65 (82.3)	29 (55.8)	3.94 (1.74–8.94)	**<0.001**
AVR	4 (5.1)	16 (30.8)	7.68 (2.49–23.68)	**<0.001**
Death from any cause	10 (12.6)	7 (13.5)	0.89 (0.23–3.45)	0.87
**Secondary outcome**				
Survival without AVR or AS progression	60 (75.9)	23 (44.2)	3.80 (1.84–7.86)	**<0.001**
AVR or AS progression	9 (11.4)	22 (42.3)	5.35 (2.33–12.28)	**<0.001**
Death from any cause	10 (12.6)	7 (13.5)	0.79 (0.22–2.82)	0.72

Primary (survival without AVR) and secondary (survival without AVR or AS progression) outcomes are shown for patients with moderate AS. HR and CI confidence interval were calculated by cox proportional-hazards model (log-rank test). A *P*-value < 0.05 (bold) was considered to indicate statistical significance. AS, aortic stenosis; AVR, aortic valve replacement; CI, confidence interval; HR, hazard ratio.

During the follow-up period 20 (15%) patients received AVR. AVR or progression of AS were observed in 31 (24%) patients (Table 3). Both AVR and AVR or progression of AS were more frequently observed in patients with ≥2 pathophysiological changes (*P* < 0.001; Table 3 and Figures 1, 2). Most patients (*n* = 17, 85%) underwent transcatheter aortic valve implantation (TAVI). Surgery was performed in three (15%) patients due to younger age or indication for coronary artery bypass grafting.

Seventeen patients (13%) died during follow-up. The number of deaths did not differ between both groups (Table 3). Cardiovascular deaths were observed in four (24%) patients: endocarditis (*n* = 1), acute pulmonary embolism (*n* = 1) and cardiogenic shock due to acute myocardial infarction (*n* = 2). Non-cardiac deaths (*n* = 13, 76%) involved pneumonia, stroke, kidney failure, intracranial hemorrhage, sepsis, and post-operative complications after non-cardiac surgery. In two patients the reason of death remained undetermined.

Multivariate analysis revealed that the presence of ≥2 pathophysiological changes was the only predictor of outcome in moderate AS (*P* < 0.001). Age, comorbidities, V_max_ > 3.5 m/s etc., were not associated with the outcome of moderate AS (Table 4). These results were consistent, irrespective of whether the endpoint was defined as survival without AVR or survival without AVR or progression of AS.

**TABLE 4:** Multivariate analysis of potential predictors of outcome in moderate aortic stenosis (AS).

Outcome	Survival without AVR		Survival without AVR or AS progression	
	HR (95% CI)	*P*-value	HR (95% CI)	*P*-value
Age > 70 years	1.01 (0.97–1.05)	0.62	1.02 (0.98–1.06)	0.39
≥2 pathophysiological changes	3.94 (1.74–8.94)	**<0.001**	3.80 (1.84–7.86)	**<0.001**
GLS > –16.5%	1.18 (0.55–2.51)	0.67	1.05 (0.53–2.06)	0.89
V_max_ > 3.5 m/s	0.54 (0.23–1.24)	0.14	0.54 (0.25–1.13)	0.10
Hypertension	1.77 (0.66–4.75)	0.27	1.31 (0.51–3.37)	0.58
CHD	0.88 (0.39–1.99)	0.77	1.09 (0.53–2.23)	0.82
LDL-cholesterol > 3 mmol/l	1.15 (0.52–2.53)	0.734	1.18 (0.59–2.37)	0.64

Multivariant analysis is shown for the primary (survival without AVR) and secondary (survival without AVR or AS progression) outcome. HR and CI were calculated by cox proportional-hazards model (log-rank test). A *P*-value < 0.05 (bold) was considered to indicate statistical significance. AS, aortic stenosis; AVR, aortic valve replacement; CHD, coronary heart disease; CI, confidence interval; HR, hazard ratio; LDL, low-density lipoprotein; V_max_, peak transvalvular velocity; GLS, global longitudinal strain.

In addition to LDL-cholesterol level, Lp(a) was available in nearly half of the patients (*n* = 57, 44%, mean 87 ± 121 nmol/l). On third (*n* = 17, 30%) had elevated Lp(a) plasma levels. Two patients (12%) with elevated Lp(a) received AVR, whereas AVR or AS progression were observed in five patients (29%). Progression rate of AVA and V_max_ per year did not differ between patients with non-elevated (AVA: –0.12 ± 0.08; V_max_: 0.19 ± 0.11) or elevated (AVA: –0.13 ± 0.06; V_max_: 0.20 ± 0.13) Lp(a) plasma levels (*P* > 0.05).

## Discussion

The main novel observation of the study is that three well-validated echocardiographic parameters which characterize AS pathophysiology, namely LVH, E/e’ and TR_Vmax_, predict clinical outcome in patients with moderate AS.

### Pathophysiology and echocardiographic findings in moderate AS

The natural history of AS is accompanied by pathophysiological changes of the left and right ventricle. A progressive narrowing of the aortic valve leads to a chronic increase of LV pressures resulting in concentric LVH. Concentric LVH induces a shift of the diastolic pressure-volume relationship followed by increasing DD with an increase of left ventricular end-diastolic filling pressure (LVEDP) which favors the development of post-capillary pulmonary hypertension (1, 4). Increases of LVMi, TR_Vmax_ and E/e’ are less frequent in moderate AS (LVMi: 66%, TR_Vmax_ ≥ 2.8 m/s: 36%, E/e’ ≥ 14: 28%) compared to patients with severe AS (TR_Vmax_ ≥ 2.8 m/s: 80%, LVMi: 79%, E/e’ ≥ 14: 69%) (10). Recently, we reported that the combination of all three parameters were detected in >50% of severe AS patients. Thus, severe AS without at least one of these pathophysiological changes is highly unlikely (10). In moderate AS, the presence of all three echocardiographic findings was only found in 18%, whereas none of those changes were found in at least 20% confirming that changes in LVMi, E/e’, and TR_Vmax_ correlate with AS progression.

Valvulo-arterial impedance has been reported to predict prognosis in moderate AS (18, 19). Despite methodological disadvantages, e.g., dependence on Doppler angle, or possible errors in BP measurement, studies have reported its usefulness as a parameter of “global hemodynamic afterload” in AS assessment (20). The mortality risk was increased 2.8-fold in patients with Z_VA_ > 4.5 mmHg⋅ml^–1^⋅m^2^ (18). Lancellotti and Magne (21) reported poor outcome in patients with moderate to severe AS with Z_VA_ > 5.5 mmHg⋅ml^–1^⋅m^2^ and reduced GLS. In the present cohort Z_VA_ was neither associated with GLS decrease nor with a poorer prognosis. However, Z_VA_ seems promising to reconcile the discordance between moderate AS and the symptomatic status. In our study, lower Z_VA_ values may indicate, that unspecific symptoms might be related to another concomitant disease. Contrary, in patients with higher Z_VA_, symptoms could reflect additive effects of moderate AS and reduced arterial compliance.

### Impact of comorbidities on AS pathophysiology

Comorbidities were mainly balanced between both groups and consistent with patient characteristics of other studies (7, 8, 10). In 2017 Genereux et al. (22) introduced a classification system describing different stages of cardiac damage being probably associated with severe AS. The extent of cardiac damage was independently associated with an increased mortality after AVR due to severe AS. As mentioned in the limitations it can be criticized that the cardiac damage detected, due to the partly serious comorbidities (e.g., moderate/severe MR, advanced chronic obstructive pulmonary disease), may not be entirely attributable to AS (22). For this reason, patients with such severe comorbidities were excluded in the present study.

In contrast, very common comorbidities, e.g., arterial hypertension (AHT) or CHD could not simply be excluded due to their high prevalence in developed countries as well as, in patients with AS. Although LV wall thickening is most commonly caused by AHT, it may also be caused by AS, edema and/or non-muscular depositions in storage diseases (23). AHT is one of the most relevant cardiovascular risk factors favoring comorbidities such as CHD and AF, which in turn may be associated with LVH (24). CHD was more prevalent in group 2 but did not reveal to be associated with the outcome of moderate AS patients. In contrast, patients with heart failure with significantly reduced LVEF and/or LVSVi were excluded because an increase in E/e’ and/or TR_Vmax_ is usually observed due to the presence of heart failure *per se* rather than due to consequences of AS in these patients.

### Predictors of outcome

Data on the outcome of patients with moderate AS have been inconsistent to some extent. Mortality rates range from 8 to 20% in moderate AS, which is consistent with the results of the present study (5, 7). In contrast, a more unfavorable prognosis with remarkably higher mortality rates was reported by other studies (6, 8). In the present study, the number of moderate AS patients who underwent AVR (15%) was significantly lower compared to other studies (19–28%) (5–8). Lower event and mortality rates in the present study could be explained by the preselected cohort, the intensive monitoring at a specialized outpatient department including optimal treatment of comorbidities and a shorter follow-up duration.

### Transvalvular velocity, pressure gradient, and valve calcification

Previous studies showed that V_max_ and AV calcification were associated with outcomes in AS. Event-free survival was significantly lower in patients with moderate and/or severe calcification (described by visual assessment) compared to patients with only mild or no calcification (2, 5). In contrast, a recent prospective study has shown that PG_mean_ and moderate-to-severe AV calcification were not associated with increased mortality in moderate AS (8). The extent of AV calcification was not assessed in the present study because visual assessment of the degree of AV calcification is associated with high interobserver variability (25). Computer tomography (CT) was not performed to avoid radiation exposure in predominantly asymptomatic patients. In addition, repeated CT examinations are difficult to integrate in the routine care of patients with moderate AS. Nevertheless, calcium scoring is recommended as a part of an integrated approach especially in AS with conflicting echocardiographic results (3, 26).

### Left ventricular ejection fraction, stroke volume, and diastolic dysfunction

Ito et al. has shown that patients (*n* = 696) with moderate AS and reduced LVEF, and/or SVi, and elevated E/e’ had a poorer prognosis, even if only E/e’ was elevated and LVEF and SVi were preserved (27). The main challenge is that patients with AS usually have many comorbidities leading to pathophysiological changes (e.g., LV hypertrophy or increase of LV filling pressure). Despite a higher proportion of patients with CHD by Ito et al. (27) the distribution of comorbidities was similar to our study and the majority of patients had preserved LVEF (>80%) or SVi ≥ 35 ml/m^2^ (>90%), respectively. Moreover, in both studies, a relevant diastolic dysfunction was considered only insofar as an increase of E/e’ was present. In general, an elevation of E/e’ can probably be attributed to ischemic or non-ischemic cardiomyopathy with reduced LVEF. However, more than 50% of the patients by Ito et al. (27) showed elevated E/e’ values although LVEF and/or SVi were normal. Similarly, patients with significantly reduced LVEF and/or SVi were excluded in the preset study. Hence, both studies suggest that especially E/e’ must be given high importance in risk stratification of patients with moderate AS. Nevertheless, more complementary data is needed in this area of moderate AS. Another study has proven that higher NT pro-BNP levels were associated with higher mortality rates in patients with moderate AS (28). In the present study no significant differences for NT pro-BNP levels were observed between both cohorts, mainly due to the smaller study population and because patients with significantly reduced LVEF and/or SVi were excluded. Although NT pro-BNP is not a specific biomarker to directly quantify AS severity, it is helpful for further risk stratification to verify cardiopulmonary congestion probably due to relevant AS (3, 28).

### LDL-cholesterol and Lp(a) plasma levels

Low-density lipoprotein-cholesterol and Lp(a) are causally associated with atherosclerotic cardiovascular diseases (29, 30). Contrary, studies found out that the natural history of AS cannot directly be influenced by medical therapy (31). In a subanalysis of the ASTRONOMER trial higher Lp(a) plasma levels (>58.5 mg/dl; >140 nmol/l) were associated with a faster progression rate of V_max_ in mild-to-moderate AS (11). We also observed slightly higher progression rates of AVA and V_max_ in patients with elevated Lp(a) plasma levels although these differences did not reach statistical significance. Presumably, this might be due to the shorter follow-up period and the small number of patients with elevated Lp(a) plasma levels in our cohort (11).

### Deformation imaging in moderate AS

Several studies have proven the prognostic value of GLS mainly by detecting subclinical LV dysfunction due to myocardial fibrosis (32, 33). Zhu et al. (33) reported significantly higher mortality rates in moderate AS with preserved LVEF and GLS > –15.2%. A difference of almost Δ –5% between both groups indicates that some patients had remarkably low GLS values. Further, patients with an GLS > –15.2% were older, had higher NT pro-BNP and more often CHD and/or myocardial infarction (33). A GLS decrease can probably be attributed to an increase of LV afterload even in moderate AS (32). Further, comorbidities and age contribute to an impairment of GLS (34, 35).

Although a GLS > –16.5% turned not out to be a predictor of outcome, a GLS decrease from baseline to follow-up indicates subclinical impairment of longitudinal LV deformation which can be attributed to AS progression. These considerations were supported by significantly lower GLS at follow-up in patients with ≥2 pathophysiological changes.

## Limitations

Although we aimed to characterize our population of moderate AS patients as well as possible, it cannot be ruled out, that pathophysiological changes (e.g., left ventricular hypertrophy) may also be proportionally caused by comorbidities, e.g., arterial hypertension. Although patients with ischemic cardiomyopathy (CHD) or patients with non-ischemic cardiomyopathy with significantly reduced LVEF (<45%) were excluded, patients with AHT could not simply be excluded due to its high prevalence. Due to the strict exclusion criteria results of the present study cannot generally be applied to all patients with AS. The data analysis deliberately focused on the distinction between moderate AS patients with <2 and ≥2 pathophysiological changes, because there were no differences between subgroups with no, only 1, 2, or 3 pathophysiological changes, respectively. Death may be a competing event for AVR. However, the number of deaths were not different between both groups and cardiovascular deaths did only occur in four patients. Further studies are needed to quantify the quality of life and physical capacity of patients with moderate AS.

## Conclusion

The presence of ≥2 pathophysiological changes (LVH, increased E/e’ and/or TR_Vmax_) is a strong predictor of outcome in patients with moderate AS. Considering these changes may be useful for risk stratification, particularly for scheduling follow-up time intervals and deciding the timing of AVR, which is still challenging based on current recommendations.

## Data availability statement

The original contributions presented in this study are included in the article/Supplementary material, further inquiries can be directed to the corresponding author.

## Ethics statement

The studies involving human participants were reviewed and approved by Universität Leipzig, Medizinische Fakultät, Ethik-Kommission, Liebigstraße, 18, Leipzig. The patients/participants provided their written informed consent to participate in this study.

## Author contributions

SS and JK: substantial contribution to the conception or design of the work, or the acquisition, analysis, or interpretation of data for the work. MM, AH, BT, and UL: drafting the work, or revising it critically for important intellectual content provide approval for publication of the content. All authors agreed to be accountable for all aspects of the work in ensuring that questions related to the accuracy or integrity of any part of the work are appropriately investigated, resolved, and approved the submitted version.

## Supplementary material

Supplementary Figure 1 Flow chart of patients with moderate aortic valve stenosis (AS) with assignment to the respective study cohorts.
