# Supplementary figures and images for: Left ventricular hypertrophy, diastolic dysfunction and right ventricular load predict outcome in moderate aortic stenosis
Source: Front Cardiovasc Med. 2023 Jan 10;9:1101493. doi: 10.3389/fcvm.2022.1101493 (PMC9871769; doi:10.3389/fcvm.2022.1101493)

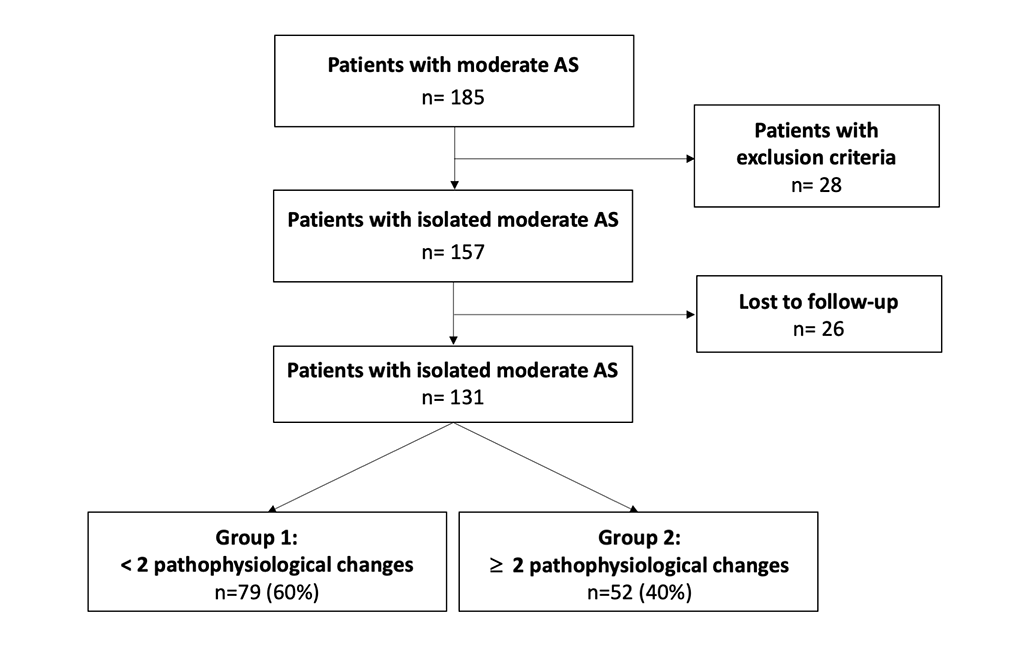

Supplement: Supplementary Figure 1 — Flow chart of patients with moderate aortic valve stenosis (AS) with assignment to the respective study cohorts. [file Image_1.TIFF]
